# P-1811. The Perfect Storm: An Analysis of the Relationship Between Texas Weather Patterns and the Incidence of West Nile Virus

**DOI:** 10.1093/ofid/ofaf695.1980

**Published:** 2026-01-11

**Authors:** Marissa Nicolas-Cagigal, Jonathan Pavia, J "Patrik" Hornak

**Affiliations:** University of Texas Medical Branch, Texas City, Texas; University of Texas Medical Branch, Texas City, Texas; University of Texas Medical Branch, Texas City, Texas

## Abstract

**Background:**

West Nile Virus (WNV) is the most prevalent mosquito-borne illness in the United States, with 20–30% of infected individuals experiencing symptomatic illness. Previous studies have suggested that temperature and precipitation strongly influence mosquito survival and therefore WNV transmission. Since 1999 the CDC has tracked U.S. cases, of particular significance is the state of Texas, which has consistently reported some of the highest case numbers. As weather patterns inevitably change it is crucial to identify environmental conditions that promote WNV transmission to inform prevention efforts.

**Figure:**
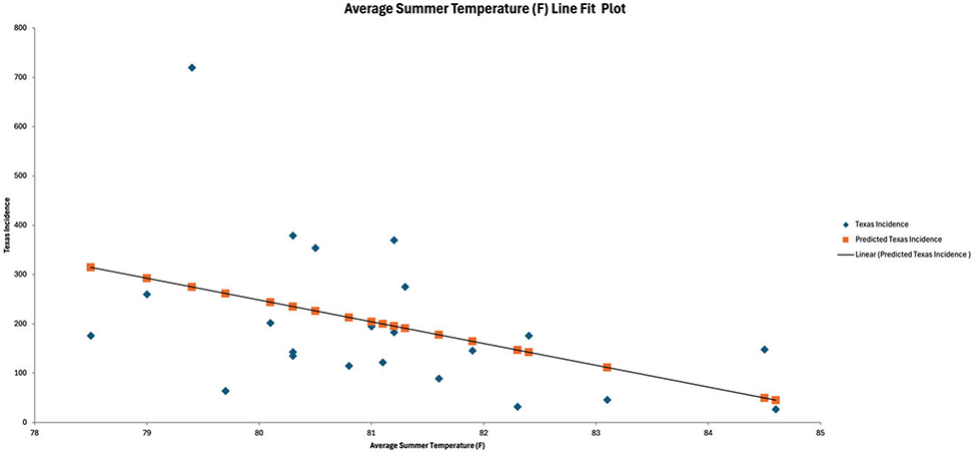
Average Summer Temperature & Incidence of West Nile Virus in Texas This figure demonstrates the negative relationship between average summer temperature and incidence of West Nile Virus in Texas

**Figure:**
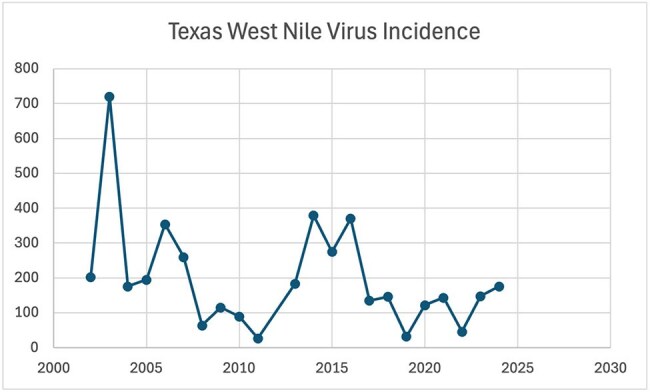
Texas West Nile Virus Incidence This figure shows the number of West Nile Virus cases in Texas that were reported to the CDC each year between 2002-2024

**Methods:**

The CDC ArboNET tool was utilized to collect WNV incidence in the state of Texas each year between 2002-2024. The National Centers for Environmental Information was utilized to collect weather data for Texas each year between 2002-2024. Weather data collected included: average summer/winter temperature and average spring/summer rainfall. Simple linear regressions were used to analyze the relationship between the incidence of WNV and Texas weather patterns. The year 2012 was excluded due to an outbreak of WNV, thus 2012 was deemed an outlier amongst the data.

**Figure:**
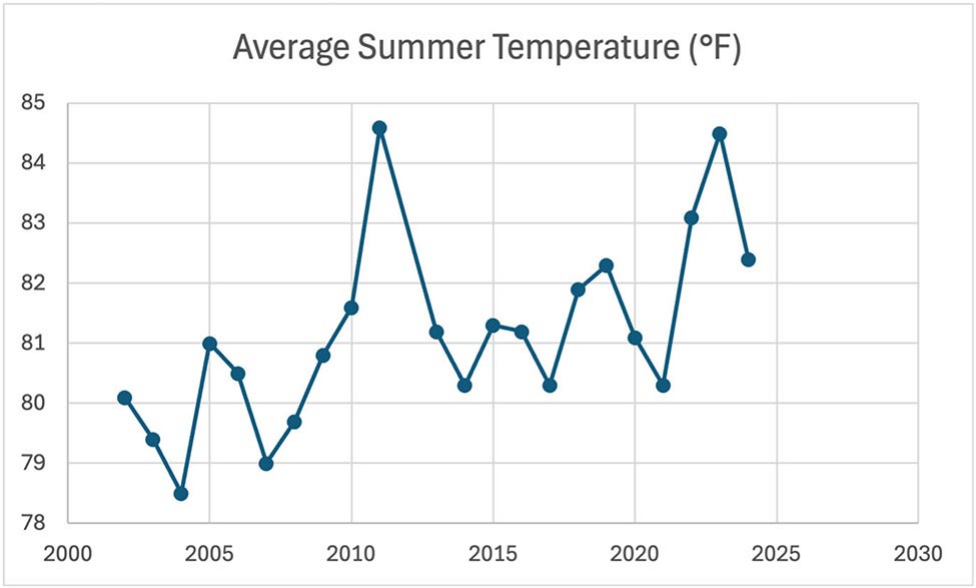
Average Summer Temperature This figure shows the average summer temperature in Texas each year between 2002-2024

**Results:**

In our analysis between the years 2002-2024, we found a significant relationship between the frequency of WNV incidence and the average summer temperatures (p = 0.038). Specifically we found for every 1°F increase in average summer temperature, the incidence of WNV decreases by ∼44.11 cases, holding all else constant. Per our analysis ∼20% of variability in WNV incidence is explained by average summer temperature alone (R^2^ = 0.198) with a 95% confidence interval of [-85.58, -2.65].

**Conclusion:**

Arthropod-borne diseases are inevitably influenced by weather patterns. Understanding the extent of this influence is of utmost importance, as it can inform effective prevention strategies. Our analysis of patterns in Texas found a statistically significant relationship between average summer temperatures and incidence of WNV. Specifically, as summer temperatures rise, the incidence of WNV decreases. The significance in relationships investigated in this study are likely underestimated due to other variables affecting transmission as well as the clinical presentation of WNV.

**Disclosures:**

All Authors: No reported disclosures

